# Pediatric congenital heart diseases: Patterns of presentation to the emergency department of a tertiary care hospital

**DOI:** 10.12669/pjms.36.3.1592

**Published:** 2020

**Authors:** Surraiya Bano, Saleem Akhtar, Uzma Khan

**Affiliations:** 1 Dr. Surraiya Bano, MBBS, MCPS, FCPS (Peds). Department of Emergency Medicine, The Aga Khan University Hospital, Karachi, Pakistan; 2 Dr. Saleem Akhtar, MBBS, MCPS, FCPS (Peds), FCPS (Peds Cardiology) Department of Pediatrics and Child Health, The Aga Khan University Hospital, Karachi, Pakistan; 3 Dr. Uzma Khan, MBBS. Department of Emergency Medicine, The Aga Khan University Hospital, Karachi, Pakistan

**Keywords:** Congenital cardiac defects, Emergency department, Pediatrics

## Abstract

**Objective:**

To observe presentation of Pediatric congenital cardiac defects to the Emergency Department (ED) of a tertiary care hospital in Pakistan.

**Methods:**

This is a retrospective chart review of patients under the age of 16 years with congenital cardiac defects presenting to the Emergency Department of Aga Khan University Hospital over a period of eighteen months, from January 2012 to June 2013. Study population was divided into two groups; first group constituted children with undiagnosed congenital cardiac defects, whereas second group constituted children with diagnosed congenial cardiac defects presented to ED. In previously diagnose cases each visit was counted as a separate encounter.

**Results:**

Out of 133 children, 44 (33.5%) were diagnosed congenital cardiac disease for the first time (Group-1) in ED, while 89 (66.5%) children were diagnosed cases of congenital heart disease (Group-2). Among Group-1; main reasons for ED visits were cyanosis, cardiac failure, murmur evaluation and cardiogenic shock where as in Group-2; main presentations were cardiac failure, hyper cyanotic spells, gastroenteritis, lower respiratory tract infection, and post-operative issues. There were total 13 deaths.

**Conclusion:**

High index of suspicion is necessary for early diagnosis and management of children with congenital heart disease in the pediatric emergency department.

## INTRODUCTION

Congenital cardiac defects are the most frequently occurring birth defect with an incidence of 4.6 to 12.2/1000 live births.^1^,^2^ Congenital heart diseases in pediatric population have a varied spectrum of presentation, especially when it comes to emergency presentation of these ailments.^3^ 0.5% to 10% of pediatric emergency room admissions are related to cardiac pathologies where congestive cardiac failure being the most prevalent of all. These affected children fall into two broad categories, the first one are those with undiagnosed cardiac defects. These defects manifest with symptoms like congestive heart failure (CHF), cyanosis, arrhythmias or cardiogenic shock. The second category belongs to children with known underlying cardiac pathologies.^4^ This category present with more generalized symptoms where the disease is precipitated by some parallel illnesses making the challenge even more arduous for emergency physicians.^5^

There is a plethora of studies describing the pathophysiology and treatment of congenital heart diseases in the medical literature. However, only a handful of studies have looked at the clinical presentation of these conditions to the Emergency department (ED).^6^,^7^ Particularly there is quite a dearth of such data in Pakistan. Therefore, this retrospective chart review of all pediatric congenital cardiac diseases was carried out in order to delineate their ED manifestations at our institution.

## METHODS

We conducted a retrospective review of the medical record of all children who visited the Emergency Department of Aga Khan University Hospital (AKUH - ED) over a period of eighteen months, from January 2012 to June 2013. Aga Khan University Hospital is a tertiary care teaching hospital, located in the city of Karachi, Pakistan, serving over a 15 million of population. The Emergency department at Aga Khan University Hospital is a 54 bedded unit both for adult and pediatric population with an annual census of 50,000 patients for both the populations. The trained Emergency Medicine faculty is available for supervision till midnight at the hospital.

The institution provides impeccable pediatric cardiology services based on three full time faculty members and a well-established fellowship program where three trainees are available round the clock. The department has its own portable echocardiography machine facilitating the diagnosis processes in the ED.

### Inclusion criteria

All the children under the age of 16 years (which is cut off age used in the pediatric ED of the study hospital) with congenital heart anomalies presenting acutely to ED were included in the study.

The study population was divided into two groups; the first one consisted of children with known cardiac defects who presented to emergency department either due to complication of their underlying cardiac problem or with some non-cardiac problem e.g. pneumonia, gastroenteritis, fever etc. The second one constituted children with undiagnosed congenital heart disease. In previously diagnose cases each emergency room visit was counted as a separate encounter.

### Data collection

Data was collected by a trained research officer. It included information related to patients’ demographics, presenting complaints, emergency department diagnoses for current admission. Moreover, in the previously diagnosed group information regarding the underlying cardiac defect, history of previous cardiac surgeries either corrective or palliative, any post-operative complications, and final disposition of patients was also taken into account. Data was entered in EpiData Version 3 and was analyzed using SPSS 19.

## RESULTS

Out of 21,000 pediatric patients seen in the emergency department during the study period, 133 children were found to have congenital heart disease which actually constituted our study population. The male to female ratio of patients was 1.4:1 (76 males and 57 females). The median age of the patients was 4.9 months with range of 0 day to 13 years. The mean length of in hospital stay was 5.6±7.3 days.

Out of 133 children, 44 (33.5%) were diagnosed as congenital heart disease (CHD) in the emergency department for the first time while 89 (66.5%) children were already known to have congenital heart disease when presented to emergency department during our study period. Patients who were newly diagnosed were more likely to present at an early age (less than 1 week) p < 0.001 (Table-I). There were total 13(10%) deaths in hospital.

**Table-I:** Patient demographics in children with congenital heart disease.

Demographics	Newly diagnosed CHD (44) n (%)	Diagnosed CHD (89) n (%)	P-Value
***Age:***			
<1 week	18(41)	02(2)	< 0.001
1-4 week	07(16)	05(6)	
4-8 week	11(25)	17(19)	
2 - 12 months	7(16)	29(33)	
1 - 5 years	00(0)	17(19)	
>5 years	1(2)	19(21)	
***Gender***			
Male	29(66)	47(53)	
Female	15(34)	42(47)	
***Cardiac lesions***			
***Left – right shunt lesions***			
VSD	13(29.5)	30(33)	0.390
ASD	02(4.5)	18(20)	<0.05
PDA	03(7)	03(3)	0.30
Endocardial cushion defect	01(2.27)	06(7)	0.26
Aorticopulmoanry window	01(2.27)	00(0.0)	
***Left sided obstructive lesions***			
Aortic stenosis	01(2.27)	01(1)	0.50
Coarction of aorta	00(2.27)	01(1)	0.66
Interrupted aortic arch	02(4.5)	00(0)	0.10
***Right sided obstructive lesions***			
Tetrogy of Fallot	01(2.27)	11(12)	<0.05
Pulmonary atresia	03(7)	05(6)	0.52
Tricuspid atresia	00(0.0)	03(3)	0.3
Transposition of great arteries	09(20.4)	03(3)	<0.01
Truncus arteriosus	02(4.5)	00(1)	0.10
Pulmonary venous abnormalities	02(4.5)	03(3)	0.53
Complex cardiac defects	04(9)	05(6)	0.34

### Characteristics of newly diagnosed CHD patients (n=44)

Eighty-two percent cases presented within 2 months of age, 41% of which presented in 1st week of their life whereas rest were between 2nd -8th weeks old.

**Fig.1:**
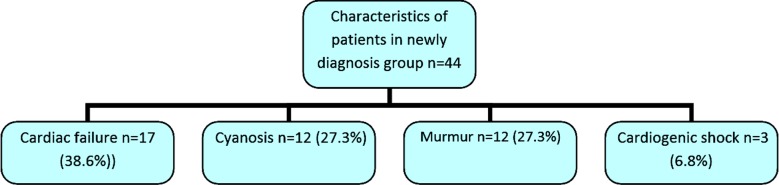
Acute manifestation of cardiac defects in group 1 (newly diagnosed).

The clinical features at the time of presentation in these patients are shown in Fig.1. Twenty-three cases (53.3%) had cyanotic while 21 patients (46.6%) had cyanotic heart disease. Eleven (25%) patients had duct dependent congenital heart defect and required prostaglandin infusion. Out of those 11 patients with duct dependent lesions; six (13.6%) patients had d-Transposition of great arteries (TGA) leading to inadequate inter circulatory mixing, two (4.5%) patients with pulmonary atresia, two had interrupted aortic arch and one had critical aortic stenosis (Table-I).

There were six deaths in newly diagnosed CHD patients. One patient died in ED had TGA with sepsis while rest in other areas of hospitals. The patients who died include two children with TGA and one each with Tausig-Bing anomaly, truncus arteriosus, interrupted aortic arch.

### Characteristics of patients with known CHD (n=89)

**Fig.2:**
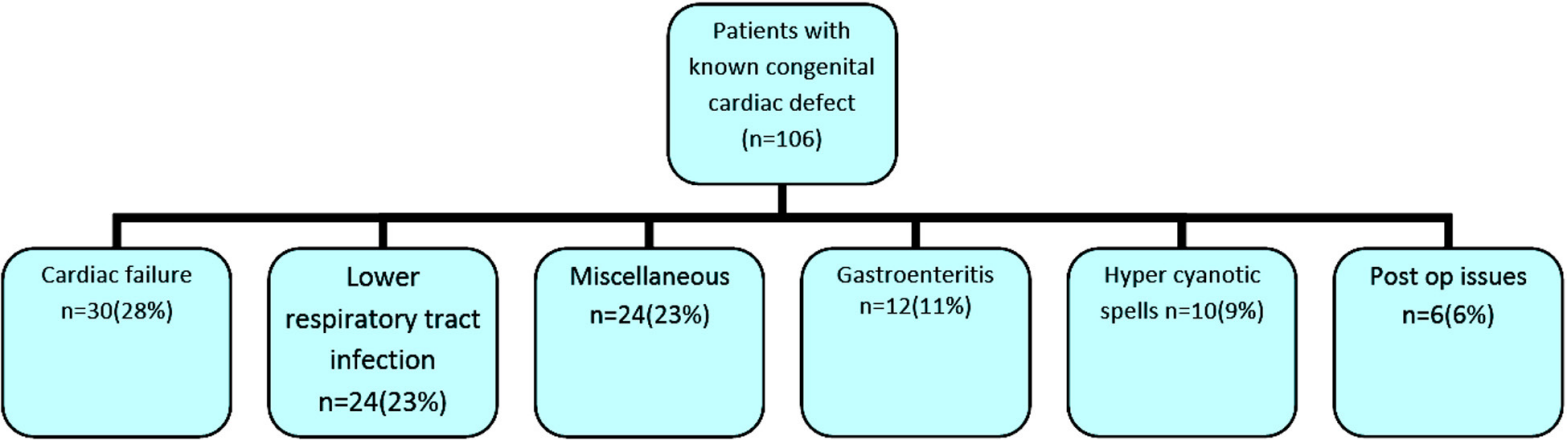
Acute manifestation of cardiac defects in group 2 (known cases).

Majority of the children presented after two months of age. Fourteen (15.7%) of known CHD children visited emergency department more than once during the study period making total of 106 visits by these 89 children. The pattern of presentation in these patients is shown in Fig.2.

These visits were mainly due to cardiac failure (28%) and respiratory tract infections (23%) and followed by gastroenteritis (11%). There were total seven (8%) deaths in this group of children, five (6%) occurred within a week of hospitalization while two (2%) occurred within four weeks of hospitalization.

## DISCUSSION

This study presents a descriptive analysis of acute manifestations of CHD in children in ED in order to familiarize with common presentations of such conditions which eventually help make precise diagnosis and accurate management of such ailments. The patients were divided into two broad categories according to existence CHD (children with known heart defects and others with new diagnosis of cardiac defects).

In both groups; cardiac failure was the main underlying diagnosis although the pathophysiology is different in both groups. Cardiac failure is a common presentation in patients with congenital heart diseases. In patients with duct dependent systemic circulation, when ductus arteriosus closes, it causes severe cyanosis or cardiogenic shock while in other cardiac defects for example; left to right shunt defects or with increased pulmonary blood flow patients will present with cardiac failure and in such patients respiratory distress could be the presenting complaints. Similar presentations were also found in studies done by Lee et al. and Savitky et al.^4^,^8^

PGE infusion was required in 25% of our patients. Lee et al. has also reported incidence of 23% duct dependent congenital heart diseases in their study.^4^ PGE infusion is critical for survival of patients with duct dependent pulmonary or systemic circulation.^9^-^13^ Any critically ill newborn in which duct dependent cardiac pathology is suspected should be started on PGE1 infusion immediately pending further evaluation.^12^,^14^-^18^

Majority of our patients in both groups had left to right shunt defects and among them VSD was the main cardiac defect. Results are comparable with other reported studies.^17^ Ventricle septal defect (VSD) is the most common congenital cardiac defect as noted in various studies and accounts for 25% of all congenital heart defects.^19^,^20^ Also in our study VSD was the most common lesion.

In Group-1 more patients presented in neonatal and infancy period with cyanotic cardiac defects where as in Group-2; age of presentation to ED was found to be more than Group-1 due to pre -dominant left to right shunt defects as described by Lee et al.^4^ Majority cyanotic heart defects are duct dependent and present early, so is the patient population in our study.

In the previously diagnosed group, congestive cardiac failure, pneumonia and Tet spell were seen in younger sub set whereas older children presented with more diverse pathologies like brain abscess, digoxin toxicity, post-operative complications, etc. Similar presenting complaints were also noted in study done by Lee et al.^4^ in which the Group-2 (already diagnosed group of patients) was further divided according to age and underlying conditions which we did not. Another study done by Savitsky et al.^8^ showed that in newly diagnosed group, most patients presented with cardiac failure where as in previously diagnosed group majority presented with cardiac failure and respiratory tract infection and worsening cyanosis (Tet spell).

### Limitations of the study

Limitation of our study consisted of it being a retrospective study (using chart review). Therefore, patients, who were discharged, transferred out or who left against medical advice could not be followed compared to patients who were admitted in our hospital.

## CONCLUSION

Emergency physicians must recognize common presentations of the common congenital cardiac defects in order to diagnose and treat these complex conditions.

### Author’s Contribution:

**SB:** Conceived, designed and writing up the paper.

**SA:** Data collection and manuscript writing, responsible and accountable for the accuracy and integrity of the work.

**UK:** Review and data analysis.
